# Supplementary material for: Reference Data for Diagnosis of Spondylolisthesis and Disc Space Narrowing Based on NHANES-II X-rays
Source: Bioengineering (Basel). 2024 Apr 8;11(4):360. doi: 10.3390/bioengineering11040360 (PMC11048070; doi:10.3390/bioengineering11040360)
Supplement: Supplementary file 1 [file bioengineering-11-00360-s001.zip › File S1 - landmark placement details.pdf]

Supplementary File S1: Details of landmark placement

The standardized landmark placement was focused on the mid-sagittal plane of each vertebral body (Figure 1), excluding any residual uncinate processes, and placing landmarks that identify the corners of the vertebral bodies prior to any osteophyte formation [(1-3)]. The posterior superior landmarks are placed so as to represent the endplate as it would appear on a mid-sagittal slice of a CT exam, rather than up at the top of any posterior ridges on the upper endplate, similar to logic described in Keynan et al and Quint et al. [(4,5)].

When the X-ray beam path through a vertebra is not perpendicular to the mid-sagittal plane of the vertebra, the left and right rims of the vertebral endplates (as seen in Figure 1), and the left and right aspects of the posterior vertebral body may be seen on the X-ray.[(3,6)] The mid-sagittal plane is assumed to be midway between the radiographic shadows of the left and right endplate rims, and midway between the radiographic shadows of the left and right aspects of the posterior wall. A similar approach was described by Quint et al.[(5)] Multiple examples of landmark placement are provided online[1] since disc space metrics calculated from landmarks are dependent on the nuances of how landmarks are placed, and it is therefore important to appreciate landmark placement details.

[1] https://www.dropbox.com/sh/qzrocrh86goxarx/AAADRXs5HoEjVdRcGIwA4beIa?dl=0

Figure 1: Details of anatomic landmark placement. Dashed lines show the estimated mid-sagittal plane of the superior and inferior endplates, identified as bisecting the radiographic shadows of the left and right sides of the endplates (yellow arrows). The red circles show the four landmarks used to measure vertebral body morphology and disc space. The red arrow points to an anterior osteophyte that is ignored when placing landmarks. The dotted green and blue lines show the anterior and posterior aspects of the vertebral body.

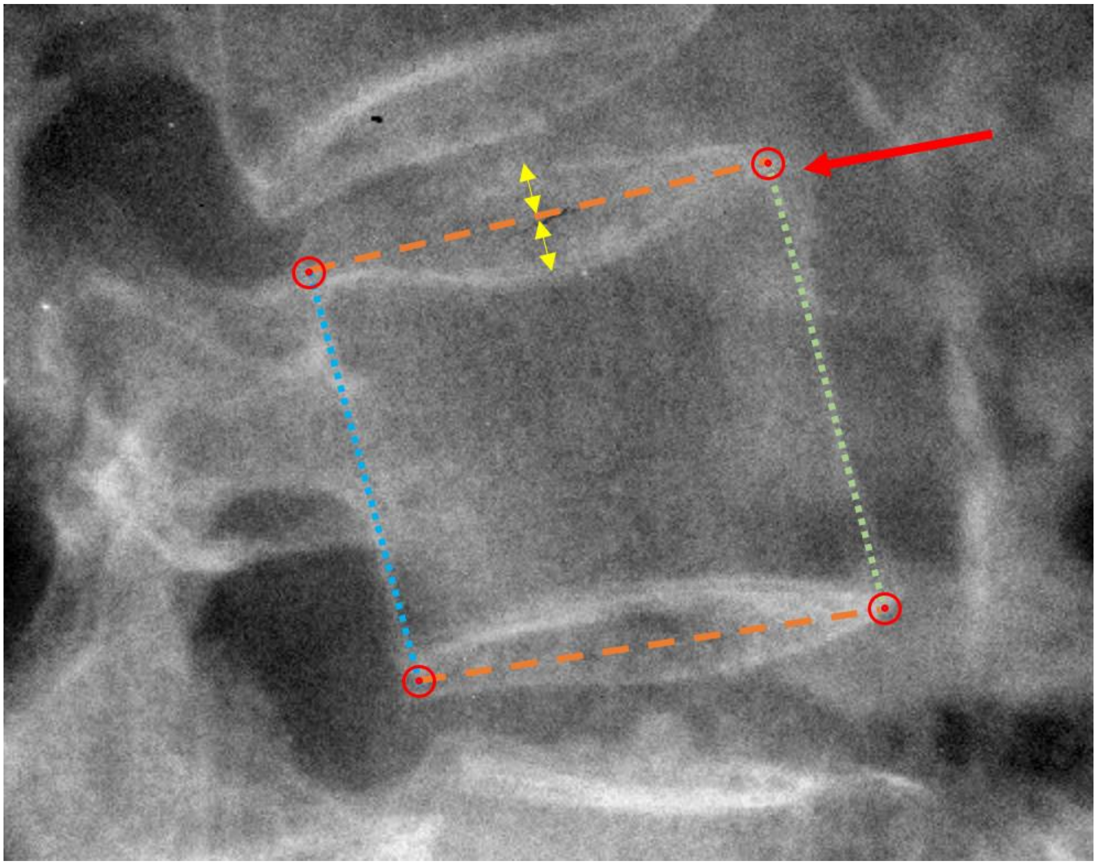

References

1. Hurxthal LM. Measurement of anterior vertebral compressions and biconcave vertebrae. Am J Roentgenol Radium Ther Nucl Med. Jul 1968;103(3):635-44.

2. Diacinti D, Guglielmi G. Vertebral morphometry. Radiol Clin North Am. May 2010;48(3):561-75.

3. Frobin W, Brinckmann P, Biggemann M, Tillotson M, Burton K. Precision measurement of disc height, vertebral height and sagittal plane displacement from lateral radiographic views of the lumbar spine. Clin Biomech (Bristol, Avon). 1997;12 Suppl 1:S1-S63.

4. Keynan O, Fisher CG, Vaccaro A, Fehlings MG, Oner FC, Dietz J, et al. Radiographic measurement parameters in thoracolumbar fractures: a systematic review and consensus statement of the spine trauma study group. Spine (Phila Pa 1976). 10.1097/01.brs.0000201261.94907.0d doi ;00007632-200603010-00025 pii Mar 1 2006;31(5):E156-65.

5. Quint DJ, Tuite GF, Stern JD, Doran SE, Papadopoulos SM, McGillicuddy JE, et al. Computer-assisted measurement of lumbar spine radiographs. Academic radiology. 1997;4(11):742-52.

6. Brinckmann P, Frobin W, Biggemann M, Hilweg D, Seidel S, Burton K, et al. Quantification of overload injuries to thoracolumbar vertebrae and discs in persons exposed to heavy physical exertions or vibration at the work-place: The shape of vertebrae and intervertebral discs—study of a young, healthy population and a middle-aged control group. Clinical Biomechanics. 1994;9:S3-S83.
