# Supplementary material for: Reference Data for Diagnosis of Spondylolisthesis and Disc Space Narrowing Based on NHANES-II X-rays
Source: Bioengineering (Basel). 2024 Apr 8;11(4):360. doi: 10.3390/bioengineering11040360 (PMC11048070; doi:10.3390/bioengineering11040360)
Supplement: Supplementary file 1 [file bioengineering-11-00360-s001.zip › File S2 - NN for osteophyes sclerosis.pdf]

Supplementary File S2: Training and validation of a neural network to identify discs with osteophytes and sclerosis

1 | INTRODUCTION

Along with demographics and health data, 7422 lateral lumbar spine X-rays were collected in a National Health survey intended to represent the entire population of the United States (the NHANES-II study). This provides an opportunity to establish normative reference data to help with objective diagnosis of disc space narrowing and spondylolisthesis. Normative vertebral body morphometry reference data have already been published.(1) In establishing reference data for normal radiographic intervertebral disc properties, it is prudent to exclude degenerated discs. That requires grading of radiographic disc degeneration. MRI exams would be optimal, but were not collected in the NHANES-II study, so only radiographic grading is possible. Manually grading every level in 7422 x-rays requires thousands of man-hours and would be tainted by errors and intra- and inter-observer variability in human assessments(2). The goal was to train and validate a neural network to automatically grade degeneration.

2 | METHODS

2.1 | Grading System

Many radiographic grading systems for disc degeneration have been used.(3) The Kellgren-Lawrence (KL) system for grading lumbar disc degeneration has been used in many studies, but like others, it requires a composite assessment of osteophytes, endplate sclerosis, and disc height loss. In practice, many levels are difficult to assign to a specific KL grade as they do not have all of the

characteristics defined for any of the grades. Exception tables are thereby needed, and application and interpretation of the grading system becomes difficult. Examples include severe disc height loss but no osteophytes or sclerosis, or large bridging osteophytes but no disc height loss. One partial solution is to separately assess disc height loss and osteophytes/endplate sclerosis. One implementation of that approach was described by Wilke et al. The Wilke et al grading system requires measurement (in millimeters) of osteophytes from lateral and AP radiographs. The magnification of the NHANES-II radiographs is unknown, and only lateral radiographs were available so measurement of osteophytes in millimeters was not possible.

The availability of reliable and near-instant automated vertebral landmarks allows for quantification of disc height loss from the landmark data. That removes the need for subjectively assessing disc height loss which is fundamentally dependent on the (unvalidated) ability of the reader to distinguish between normal and abnormal disc heights. It then remains only necessary to assess for osteophytes and endplate sclerosis. A wide range of osteophyte formation patterns have been described.(4, 5, 6, 7, 8, 9, 10) Since osteophytes frequently form on the left and right sides of vertebral bodies(5), in addition to anteriorly and posteriorly, it can be difficult, with only a lateral radiograph, to distinguish between true endplate sclerosis and lateral osteophytes. Presumably, lateral osteophytes are as clinically important as anterior osteophytes. There are no validated guidelines, for using lateral radiographs alone, to support distinguishing between lateral osteophytes, endplate sclerosis, and endplate defects such as Schmorl's nodes or healing/healed fractures. Thus, all of these phenomena will be collectively described as abnormal ossification.

With consideration of the issues discussed above, and partially based on the KL grading system, a five grade system was implemented for abnormal ossification in the region of the endplates, as described in table 1. Both the inferior endplate of the cranial vertebra and the superior endplate of the caudal vertebra are included in the grading. Osteophyte formation anteriorly, laterally, or posteriorly is

intended to be included as well as any apparent endplate sclerosis. No attempt was made to distinguish between traction and claw type osteophytes(11), since in practice, a wide range of osteophytes were observed and only two osteophyte categories would not be sufficient. Note that disc space narrowing is not considered in this assessment – that will be assessed separately from automated landmarks. Multiple examples of each grade are provided in Figure 1.

Table 1: Abnormal lumbar disc space ossification grading system. Both the superior endplate of the caudal vertebra and the inferior endplate of the cranial vertebra are included in the assessment. Osteophyte/ sclerosis at either the anterior or posterior aspects of the endplate, or on the right or left sides of the endplates are factored into the assessment.

Grade	Label	Description
1	None	There is no evidence of any osteophyte formation, endplate sclerosis, or other abnormal ossification patterns
2	Possible	Osteophytes or endplate sclerosis may be starting to form, but this is not definitive. This can appear as either a small protrusion at one edge of the endplate, increased density at one corner of the endplate, or fuzziness along the endplate that could be due to initial osteophyte formation on the left or right sides of one of the endplates
3	Definite	Definitive evidence of early stage osteophytes or sclerosis that has not progressed to a moderate extent
4	Moderate	Solitary osteophytes, sclerosis, or other abnormal ossification has progressed to a moderate extent
5	Severe	Large and/or multiple moderate sized osteophytes, endplate sclerosis, or other abnormal ossification is clearly evident

2.2 Training/Validation Images

The lateral lumbar radiographs from the NHANES-II study had been previously obtained from a publicly available source. There were 7415 useable NHANES-II lumbar radiographs. Disc degeneration was graded for 454 of those x-rays for use in training and validation of the neural network. In addition, to include lateral lumbar X-rays from additional sources, disc degeneration was also assessed for 1813 fully anonymized lateral lumbar radiographs from several other, IRB approved projects previously analyzed at Medical Metrics, Inc. Data were split 80% for training and 20% for validation. All visible discs (L1-L2 to L5-S1) in each X-ray were graded. Results from an early version of the neural network (trained on a small proportion of the data) were used to help select x-rays for grading where at least two levels in the spine were degenerated. This was done to get a similar proportion of degenerated and non-degenerated levels to avoid unbalanced final neural network training.

To facilitate grading, cropped images of each of the disc spaces from L1-L2 to L5-S1 were prepared using the vertebral landmarks produced by a pipeline of neural networks and coded logic. The cropped images were standardized such that:

- The geometric center of the disc space was centered in the image
- The disc bisectrix was horizontal in the image.
- The average of the endplate widths of the superior endplate of the inferior vertebra and inferior endplate of the superior vertebra was 50% of the 300 pixel width of each cropped disc image. Thus, any osteophytes in a region that was 50% of the endplate width anterior or posterior to the endplate were included.
- The height of each cropped disc image was equal to the average endplate width. Images were padded with a black box above and below the disc image to get a 300x300 pixel image for neural network training/validation

- Preprocessing was applied to the cropped image including: rescaling of pixel intensities to use the full range of pixel values, sigmoidal contrast enhancement, and histogram equalization.

The assessment of abnormal ossification was facilitated by a custom program that sequentially displayed each disc space to the reader and required only the selection of a single grade through a graphical user interface (or pressing "q" to quit a session of grading). The custom program kept track of what had already been graded and presented only ungraded levels upon restart. Images were displayed magnified to a 30 x 30 cm window on a high-quality monitor. Note that this allowed for more detailed scrutiny of the disc space than would be typical in a clinical assessment of radiographic disc degeneration.

2.3 | Description of the neural network

The following elements were part of the neural network that was trained and validated to grade osteophytes/sclerosis:

- Backbone: ResNet V2 50x1 BitM (https://arxiv.org/pdf/1912.11370.pdf)
- FC Layer: CORAL Layer for Rank-Consistent Ordinal Regression (https://arxiv.org/pdf/1901.07884.pdf)
- Regularization: Weight Decay, Spectral Decoupling Loss (SD Loss) (https://arxiv.org/abs/2011.09468)
- Training Scheduler: 1-Cycle Training Schedule(https://fastai1.fast.ai/callbacks.one_cycle.html)
- Optimizer: SAM (https://arxiv.org/pdf/2010.01412.pdf)

- Pooling: Adaptive Average Pooling
- Integrated Z-Scoring of Inputs: Z-scoring of image is done in the forwards pass of the model, so there is no need to z-score the image before being passed as an input
- Optuna was used for hyperparameter tuning (https://optuna.org/)

The training data were augmented by creating additional vertebral landmarks that had a normal distribution of noise added to the original x and y coordinates of the centroid (+/- 4%), to the crop angle (+/- 6 degrees), and to the FOV / Endplate Width used (+/- 10%). The additional vertebral landmark data were used to create additional disc space images for training.

For the purposes of establishing normative disc height and spondylolisthesis data from the NHANES-II x-rays, it was necessary to only classify each disc as non-degenerated (to be included in defining normal) or degenerated (to be excluded). Therefore, grades 0 and 1 were combined to classify discs that were non-degenerated, and all higher grades were classified as degenerated. The neural network produced a degeneration score for each disc image that ranged from -4.1 to 6.6 and the score was calibrated such that any score > 0 was classified as having osteophytes/sclerosis.

3| Results and Discussion

Table 2 summarizes the validation results from the neural network (percentages are relative to all levels graded). The kappa score between the manual and automated grading was 0.78. The automated grade was 87% sensitive and 91% specific to the manual grade.

Table 2: Neural network validation results

Human Classification	Automated Classification	
	Not Degenerated	Degenerated
Not degenerated	49.0%	4.9%
Degenerated	6.2%	39.8%

A very diverse range of osteophytes and endplate sclerosis was encountered during manual grading. Some levels were easy to manually grade while others were difficult as they were borderline between the high-end of one grade and the low-end of the next grade, the level was poorly imaged, or there was an unusual pattern of osteophytes or sclerosis. In some images, the disc space could barely be discerned and in others, there was confounding artifacts (overlying ribs, blood vessel plaque, bowel gas, blatant pixelation, zippers, etc). Unless the disc space was almost completely unrecognizable, a grade was manually assigned. It may have been better to have used a low-tolerance for poor image quality and thereby include an "ungradeable" class, though that would result in a potentially sub-optimal proportion of ungradeable cases in research studies and clinical practice. Manual checking of randomly selected levels found no instances of definitive AI errors. Discrepancies between the manual grading and the automated grading were always instances of borderline cases.

There was a highly significant difference in average disc space narrowing between levels graded as having osteophytes/sclerosis versus those without ($P<0.0000$). However, there were levels not graded as having osteophytes/sclerosis that did have disc space narrowing or spondylolisthesis. Those levels were excluded from establishing reference data for normal disc properties using disc space narrowing and spondylolisthesis metrics.

It is possible that if MRI were available, early stage degeneration would have been found at some levels that had not progressed to radiographically evident degeneration, although there is conflicting evidence on whether MRI or X-rays are best for detecting early stage degeneration. (12, 13) Nevertheless, there was high confidence that the automated grading found most if not all levels that were definitively degenerated.

The automated osteophyte/sclerosis grading was obtained for all of the NHANES lumbar X-rays. Table 1 summarizes the proportion of levels found to have osteophytes/sclerosis by the neural network. The difference between sexes is likely due to the older ages of the females (63.3±6.4 vs 50.9±15.3).

Table 1: The percent of levels in the NHANES-II lumbar spine X-Rays that were found to have osteophytes/sclerosis by the neural network.

	% with Osteophytes/Sclerosis	
Level	Males	Females
L1L2	28.6	36.4
L2L3	34.7	41.6
L3L4	39.4	42.2
L4L5	32.6	34.7
L5S1	18.4	23.2

With logistic regression, age (odds ratio 1.1), sex (odds ratio 0.58 – osteophytes/sclerosis less likely in males), and BMI (odds ratio 1.03) were all significantly ($P<0.000$) associated with the automated osteophyte/sclerosis score though the overall predictability based on these parameters was low ($R^2=0.13$). Race and nation or origin were not significant ($P>0.3$).

Figure 1: Examples of disc images classified by the neural network. Top row: Grade 0 = no osteophytes/sclerosis (OS). Second row: Grade 1 = possible OS. Third row: Grade 2 = definite OS. Fourth row: Grade 3 = moderate OS. Bottom row: Grade 4 = severe OS.

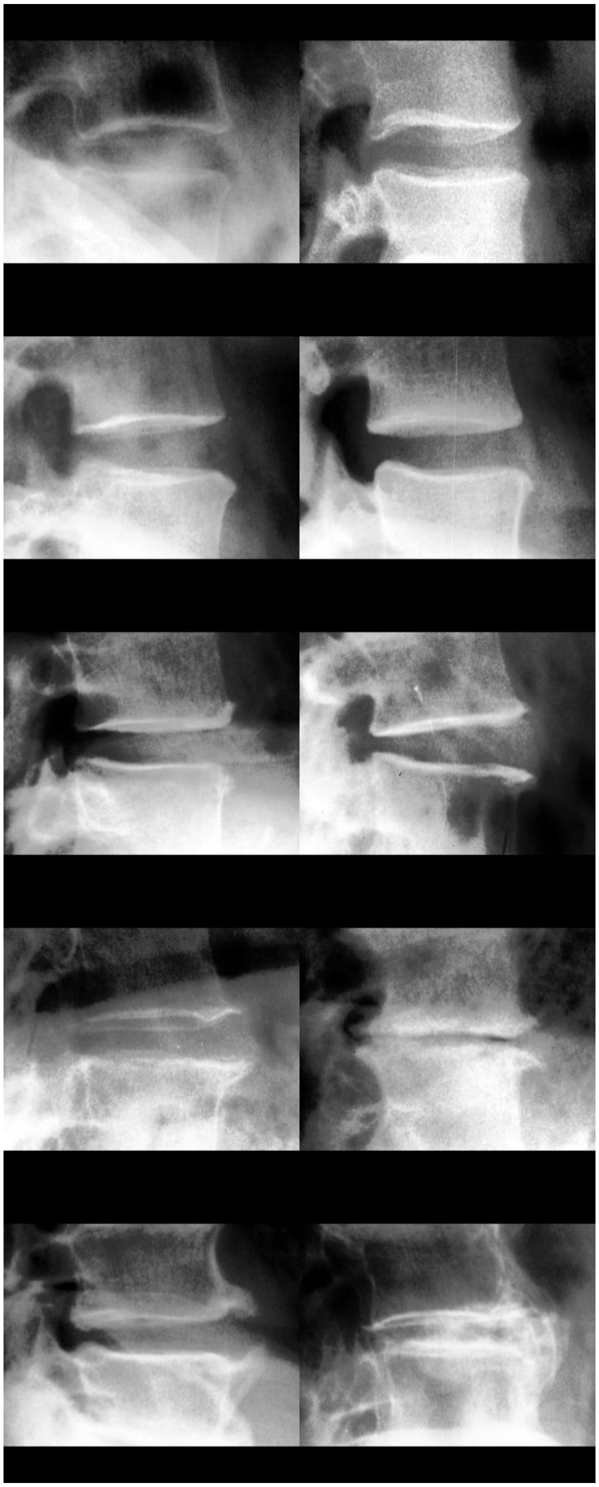

References

1. Hipp JA, Grieco TF, Newman P, Reitman CA. Definition of Normal Vertebral Morphometry Using NHANES-II Radiographs. JBMR Plus. 2022;6(10):e10677.
2. Chen X, Sima S, Sandhu HS, Kuan J, Diwan AD. Radiographic evaluation of lumbar intervertebral disc height index: an intra and inter-rater agreement and reliability study. Journal of Clinical Neuroscience. 2022;103:153-62.
3. Tan TL, Borkowski SL, Sangiorgio SN, Campbell PA, Ebramzadeh E. Imaging criteria for the quantification of disc degeneration: A systematic review. JBJS reviews. 2015;3(2):e2.
4. Kasai Y, Kawakita E, Sakakibara T, Akeda K, Uchida A. Direction of the formation of anterior lumbar vertebral osteophytes. BMC musculoskeletal disorders. 2009;10(1):1-6.
5. Nathan H. Osteophytes of the vertebral column: an anatomical study of their development according to age, race, and sex with considerations as to their etiology and significance. The Journal of Bone and Joint Surgery. 1962;44(2):243.
6. Öğrenci A. Bone Protrusion That We Should be Aware of: Foraminal Osteophytes; Classification and Surgical Results. Haseki Tip Bulteni. 2018;56(4):299.
7. Van der Merwe AE, Işcan M, L'Abbè EN. The pattern of vertebral osteophyte development in a South African population. International Journal of Osteoarchaeology. 2006;16(5):459-64.
8. Heggeness MH, Doherty BJ. Morphologic study of lumbar vertebral osteophytes. Southern Medical Journal. 1998;91(2):187-9.
9. Pye SR, Reid DM, Lunt M, Adams JE, Silman AJ, O'Neill TW. Lumbar disc degeneration: association between osteophytes, end-plate sclerosis and disc space narrowing. Ann Rheum Dis. 2007;66(3):330-3.
10. Quinnell RC, Stockdale HR. The significance of osteophytes on lumbar vertebral bodies in relation to discographic findings. Clinical Radiology. 1982;33(2):197-203.
11. Macnab I. The traction spur. An indicator of segmental instability. J Bone Joint SurgAm. 1971;53(4):663-70.
12. Benneker LM, Heini PF, Anderson SE, Alini M, Ito K. Correlation of radiographic and MRI parameters to morphological and biochemical assessment of intervertebral disc degeneration. European spine journal : official publication of the European Spine Society, the European Spinal Deformity Society, and the European Section of the Cervical Spine Research Society. 2005;14(1):27-35.

13. Frobin W, Brinckmann P, Kramer M, Hartwig E. Height of lumbar discs measured from radiographs compared with degeneration and height classified from MR images. EurRadiol. 2001;11(2):263-9.
