# Supplementary material for: Reference Data for Diagnosis of Spondylolisthesis and Disc Space Narrowing Based on NHANES-II X-rays
Source: Bioengineering (Basel). 2024 Apr 8;11(4):360. doi: 10.3390/bioengineering11040360 (PMC11048070; doi:10.3390/bioengineering11040360)
Supplement: Supplementary file 1 [file bioengineering-11-00360-s001.zip › File S3 - spondyindex from flex ex.pdf]

Supplementary File S3: Predicting normal sagittal plane offset for use in a diagnostic test for spondylolisthesis

1 | Introduction

In research and in clinical practice, it may be valuable to be able to predict normal Sagittal Plane Offset (SPO) for each specific level such that the abnormal SPO (spondylolisthesis) can be objectively diagnosed. It was hypothesized that normal sagittal plane offset can be predicted from level, disc heights, and other disc metrics. Observing cases where one vertebra appears wider or narrower than the adjacent vertebra, it was hypothesized that this endplate width discrepancy would influence SPO measurements. To test this hypothesis, an EndPlate Width Ratio (EPWR) was calculated as the anterior-posterior width of the inferior endplate of the superior vertebra divided by the superior endplate width of the inferior vertebra. This ratio was included in an equation to predict normal SPO.

2 | Methods & Results

To understand the variables that can be used to predict normal SPO, multivariate analysis of variance was used with the NHANES lumbar data, excluding cases with osteophytes/sclerosis. Multiple ANOVA tests were performed using different combinations of metrics. The best and most practical combination was level, disc area, endplate width ratio, and anterior and posterior disc heights (R^2=0.94). Regression analysis was then used to obtain an equation that can be used to predict ASPO from other disc metrics.

The average difference between the predicted ASPO and the actual ASPO, using only the trimmed NHANES-II data, was 0.0 % endplate width (std dev 0.84). The differences ranged from -4.1 to

6.7 % endplate width. Including all of the NHANES-II data, the difference between the predicted and actual ASPO was 0.02±1.8 (range -45 to 17 % EPW). A standardized metric was then calculated, using as the denominator a point prediction of the error – the Standard Error of the Forecast (STDF). The numerator is the difference between the predicted and actual ASPO. That metric is referred to as the Spondylolisthesis Index (SI). If it is between -2 and +2 it is considered statistically within normal limits. Review of images with high values of the SI revealed that when SI is > 3, then spondylolisthesis is clearly evident, but between 2 and 3, it is more subtle.

The NHANES-II radiographs were all obtained with subjects side-lying. There was relatively little variation in L1-S1 angle between subjects (51.3±12.8), compared to what can be expected with flexion-extension studies (35.1±33.1) in the study that will be described below). Some symptomatic patients may stand with forward flexion due to pain, or may otherwise have unusual lordosis, and it would be valuable to objectively detect abnormal SPO in those patients. Spondylolisthesis may also sometimes be assessed from flexion and extension (FE) radiographs in clinical practice and research studies[1]. Therefore, Anterior SPO (ASPO), Posterior SPO (PSPO) and Centroid SPO (CSPO) were also analyzed from flexion-extension radiographs of asymptomatic volunteers. Figures 1 and 3 in the paper that references this appendix provide diagrams illustrating these SPO metrics.

Some (161) of the asymptomatic volunteer FE radiographs were described and analyzed in a prior publication.[2] An additional 220 FE radiographs were also analyzed. The first 161 FE studies had been obtained using a seated protocol [2], while the second 220 had been obtained with an upright protocol using a standard walker for support.[1] Each of the 220 subjects watched a short training video before the

[1] https://youtu.be/YDcMMZdc7dc

X-rays were collected[2]. All 381 subjects consented to the IRB approved studies, were asymptomatic, and had never had medical treatment for a back disorder.

The SI derived using the NHANES data was applied to the asymptomatic FE studies. With an abnormal SI defined as < -2 or > 2, 33% of the levels in the asymptomatic population were classified as abnormal. That is clearly too high and is likely explained by insufficient representation of high and low disc angles in the NHANES data. Therefore, a SI was derived using the asymptomatic FE studies.

The FE studies were analyzed using the same series of neural networks and coded logic (Spine CAMP™, Medical Metrics, Inc., Houston, TX) as with the NHANES-II X-rays. A neural network that was trained to identify definitive osteophytes and/or sclerosis was used to find levels with clear signs of degeneration (Appendix 2). Levels were excluded from calculation of a SPO prediction equation if the neural network found osteophytes/sclerosis OR there was substantial disc height loss OR there was substantial spondylolisthesis. Regression analysis was used to find an equation that was highly predictive of normal SPO. (Stata ver 15, College Station, TX).

Based on the neural network grading of osteophytes/sclerosis, 17.2% of levels were excluded. An additional 0.35% of levels were excluded because of spondylolisthesis or disc space narrowing that did not have osteophytes/sclerosis. In summary, 17.6% of levels were excluded from establishing an equation to predict normal SPO from disc metrics obtained from lumbar flexion-extension X-rays.

Analysis of variance revealed that the combination of level, ADH, PDH, EPWR, and disc area predicted 94% of the variation in ASPO in the FE X-rays. Adding disc angle had minimum impact on the R^2 so it was not included in the model. Adding PSPO to the multi-variate analysis of variance increased

[2] https://youtu.be/KaEQP57qWgU English language version

https://youtu.be/Day_wvEG-yI Spanish language version

the R^2 to 0.98, though using PSPO to help predict ASPO was judged inappropriate. A regression equation was developed for predicting normal ASPO from ADH, PDH, EPWR, and Disc Area. Using the data for levels with no osteophytes/sclerosis or spondylolisthesis, Equation 1 was established.

Equation 1: Predicted ASPO = C1 + ADH * C2 + PDH * C3 + EPWR * C4 + Disc Area * C5 + C6

Where:

C1 = 0.27 if L1-L2; -0.05 if L2L3; 0.49 if L3L4; 1.22 if L4L5, 0 if L5S1
C2 = 2.6
C3 = 3.1
C4 = 1373
C5 = -565
C6 = -137

As a check of the regression equation, the predicted ASPO was linearly correlated to the actual ASPO for the normal disc levels (R^2 = 0.94).

Equation 1 was then used to predict ASPO in the NHANES study. The average difference between the actual ASPO and the predicted ASPO, using only the trimmed NHANES-II data, was -0.19 % endplate width (std dev 1.16). The differences ranged from -5.1 to 4.1 % endplate width. Including all of the NHANES-II data, the difference between the actual ASPO (from Equation 1) and predicted was -0.12±2.11 (range -17.5 to 47). The predicted ASPO using equation 1 was strongly (R^2 = 0.78) and linearly (slope = 1) correlated to the actual ASPO. A stronger correlation would not be expected, since the predicted ASPO is the predicted **normal** ASPO, and actual ASPO in the NHANES-II study is not always normal. It is the difference between the actual and predicted (normal) ASPO that could be of clinical value. This supports the potential value of the ASPO prediction equation from the asymptomatic flexion-extension X-Rays. Further research will be required to determine which metric is best for classifying SPO

as normal versus abnormal: the standardized ASPO, PSPO, or CSPO metric from the NHANES-II study, or the prediction equation derived from the flexion-extension studies of asymptomatic volunteers. The equation from the asymptomatic volunteer flexion-extension appears to have broader applicability. These results also support the hypothesis that it is necessary to account for disc metrics when predicting what normal SPO should be, particularly if there is a lot of flexion or extension.

ASPO predicted using the flexion-extension X-rays can also be expressed as a standardized metric (difference from average normal). Since the predicted ASPO is calculated from an equation, a different approach to standardization is used. To obtain the denominator needed to calculate a standardized predicted ASPO, the standard error of the forecast was calculated after the regression. The standard error of the forecast is the standard error for a point on the curve. There was minimal difference between levels, so one average value for all levels was used (1.41). A standardized SPO that accounts for disc area, EPWR, and disc heights was then calculated as the actual ASPO minus the predicted ASPO divided by the standard error of the forecast. This is referred to as the spondylolisthesis index (SI) to distinguish it from the other SPO metrics described in the NHANES-II study.

When this SI was applied to the NHANES data, excluding levels with osteophytes/sclerosis or abnormal disc metrics, it averaged -0.13±0.82, range -3.6 to 2.9. Only 0.5% of levels had a SI > 2. Including all levels in the NHANES lumbar x-rays, the SI averaged -0.085±1.5 with a range of -12 to 34. An SI > 2 was found in 4.2% of all levels. In review of NHANES X-rays, it was observed that when the SI was > 3, spondylolisthesis was clearly evident but between 2 and 3, it is more subtle. A large negative SI was more difficult to interpret. This is because the SI is the difference between the actual and predicted SPO. If the actual SPO is negative and the SI is a negative number, the SI informs that the actual SPO is less negative than predicted, but this may not necessarily appear as an anterior spondylolisthesis. For this reason, a posterior spondylolisthesis index is also calculated using similar methods. Equation 2 and coefficients are provided below. Thus, an anterior SI > +2 can be used to diagnose anterior

spondylolisthesis and a posterior SI > +2 can be used to diagnose retrolisthesis. Large negative anterior SI or posterior SI values can thereby be ignored.

Equation 2: Predicted PSPO = C1 + ADH * C2 + PDH * C3 + EPWR * C4 + Disc Area * C5 + C6

Where:

C1 = 0.24 if L1-L2; 0.63 if L2L3; 0.11 if L3L4; -0.78 if L4L5, 0 if L5S1

C2 = -1.7

C3 = -2.0

C4 = -9.19

C5 = 370

C6 = 10.1

References

1. Mummaneni PV, Bisson EF, Kerezoudis P, et al. Minimally invasive versus open fusion for Grade I degenerative lumbar spondylolisthesis: analysis of the Quality Outcomes Database. *Neurosurgical focus*. 2017;43(2):E11.

2. Staub BN, Holman PJ, Reitman CA, Hipp J. Sagittal plane lumbar intervertebral motion during seated flexion-extension radiographs of 658 asymptomatic nondegenerated levels. *Journal of neurosurgery Spine*. Dec 2015;23(6):731-8. doi:10.3171/2015.3.SPINE14898
