# Supplementary material for: Reference Data for Diagnosis of Spondylolisthesis and Disc Space Narrowing Based on NHANES-II X-rays
Source: Bioengineering (Basel). 2024 Apr 8;11(4):360. doi: 10.3390/bioengineering11040360 (PMC11048070; doi:10.3390/bioengineering11040360)
Supplement: Supplementary file 1 [file bioengineering-11-00360-s001.zip › File S4 - Errors from OOP.pdf]

Supplementary File S4: Analysis of errors due to out-of-plane imaging

1 | Introduction

Understanding the sources of errors in measurements helps to understand when reported metrics should be interpreted with caution, or not relied upon at all. This understanding can also help with automated systems for producing metrics where rules can be established to either add a caution note in a report or to abstain from reporting data.

2 | Materials and Methods

To document expected variability in the metrics due to variations in radiographic projections that could occur in clinical practice, digitally reconstructed radiographs (DRR) were used. Thin-slice (1 mm or less), anonymized computed tomography exams of the lumbar spine of 26 individuals were interpolated to 0.2 mm isotropic resolution. The interpolation was done to achieve higher resolution DRRs. The three-dimensional (3D) coordinates of the four anatomic landmarks described in Supplementary Appendix 1, Figure 1 were manually digitized in 3D in the mid-sagittal plane of each vertebra from L1 to S1. Landmark position was identified using intersecting axial, coronal, and sagittal slices. Landmarks were thus placed in the mid-sagittal plane at clear and well-defined locations. The image processing and landmark placement were completed using Slicer 3D.[1] The disc metrics described in the paper were calculated directly from the 3D landmark coordinates and used as the "gold standard" to assess accuracy of measurements from the DRRs.

Custom python code was developed to create 2D, lateral, digitally reconstructed radiographs from the 3D CT data using the Plastimatch DRR program[2]. A 40 inch source-to-image distance was used for all

X-rays. The base x-ray was centered on the centroid of the L3 vertebra (this is the baseline isocenter). The distance between the center of L3 and the image was set to ½ the estimated frontal plane skin-to-skin width of the hips. The projection matrices used by Plastimatch DRR to create the simulated X-rays were then used to calculate, from the known 3D coordinates, the precise coordinates of each landmark on each 2D simulated X-ray. In addition to the baseline DRR, forty additional DRRs were generated with the variations described below. There were thus 41 sets of measurements for every disc level (L1-L2 to L5-S1) included in the field-of-view of the CT exam for each of the 26 independent CT exams.

- random beam tilts of between ± 10 deg in each of the sagittal and axial planes
- random displacements from the baseline isocenter of ± 2 endplate widths in the AP and LR directions, plus ± 3 endplate width shifts in the cranial-caudal direction
- random image rotations between ± 24 deg

These variations resulted in a wide range of radiographic projections intended to represent the variations encountered in clinical practice, and represent a digital version of the methodology used in earlier studies that addressed the effect of radiographic projection on disc height measurements. [3-5] The landmarks in every image were precisely calculated so all 2D landmark coordinates corresponded to the true 3D coordinates. This allows for documenting the effect of errors in vertebral morphology metrics that are due to radiographic projection, without any uncertainty in landmark placement. These precisely calculated landmarks were analyzed to obtain the disc metrics described in the paper.

Although the coordinates of landmarks in every simulated X-ray were precisely calculated based on geometry, the quality of the simulated radiographs was below clinical standards, did not include the field-of-view normally found in a lateral spine X-ray, and were therefore not appropriate for use toward validating the accuracy of neural networks in obtaining landmarks. It should also be appreciated that the simulated radiographs with no beam tilt or offset cannot be assumed to be the optimal radiograph,

since not all CT exams were obtained with perfectly oriented spines and some spines had frontal plane curvatures in the lumbar spine.

3 | Results

There were 5043 measurements for each of the disc metrics representing a wide range of variations in radiographic projection. Table 1 provides the mean, median, and 95th percentile errors in the metrics. These are errors solely due to variability in radiographic projection with no error in landmark digitization. Standardized metrics (units of Std Dev from average normal) are reported in Table 1 to facilitate interpretation of the data and allow for pooling of data for all levels. For example, the median error in ASPO, due to random variations in radiographic projection, was 0.19 std dev from average ASPO in healthy discs. The maximum error in ASPO that occurred due to variations in radiographic projection was 1.12 std dev.

4 | Discussion

In general, errors due to variability in radiographic projection are small. However, large errors can occur with severe out-of-plane imaging. Disc angles were the least effected, followed by disc heights and then SPO. The worst errors were found in CT exams of scoliotic spines (eg Figure 1). With the spine in Figure 1, optimal imaging of the L2L3 level would require substantial beam tilt, and that amount of beam tilt would yield poor quality imaging of the L4L5 level.

5 | Conclusion

This experiment documents the need for caution when interpreting sagittal plane disc and SPO metrics when the radiographic projection is substantially out-of-plane, since errors can be much larger

than average. The images can be out-of-plane both with respect to the frontal and axial planes. A method to determine when there is too much out-of-plane is challenging. This requires both a definition of how much error is too much, and a method to detect out-of-plane that would result in too much error. Neural networks can likely be trained to recognize excessive out-of-plane once error tolerances are defined.

Table 1 : Descriptive statistics to help assess the effect of radiographic projection on error in the metrics. The absolute error for each metric was measured by comparing metrics from 2D simulated radiographs to the true measurement made from 3D data. Errors are reported using standardized (“z”) scores in units of std devs from average in healthy discs. 5043 measurements were available for each metric (26 CT exams * 4 to 5 levels * 41 variations in radiographic projection).

Variable	Mean	Median	95th %
Standardized anterior disc height	0.23	0.12	0.83
Standardized posterior disc height	0.19	0.1	0.68
Standardized average (of anterior and posterior) disc height	0.25	0.13	0.91
Standardized disc angle	0.14	0.07	0.45
Standardized anterior sagittal plane offset	0.32	0.19	1.12
Standardized posterior sagittal plane offset	0.25	0.14	0.92
Standardized ventral disc height	0.22	0.12	0.74
Standardized dorsal disc height	0.17	0.1	0.63
Standardized mid-plane disc angle	0.12	0.09	0.34
Standardized centroid sagittal plane offset	0.63	0.41	2.02
Anterior spondylolisthesis index	0.28	0.16	0.94
Posterior spondylolisthesis index	0.23	0.14	0.74

Figure 1: Three-dimensional reconstruction of one of the spines in the simulated X-ray study. Considering how a lateral radiograph of the lumbar spine would appear, helps to understand how radiographically apparent disc heights and angles could change as the x-ray beam angle changes in the frontal plane.

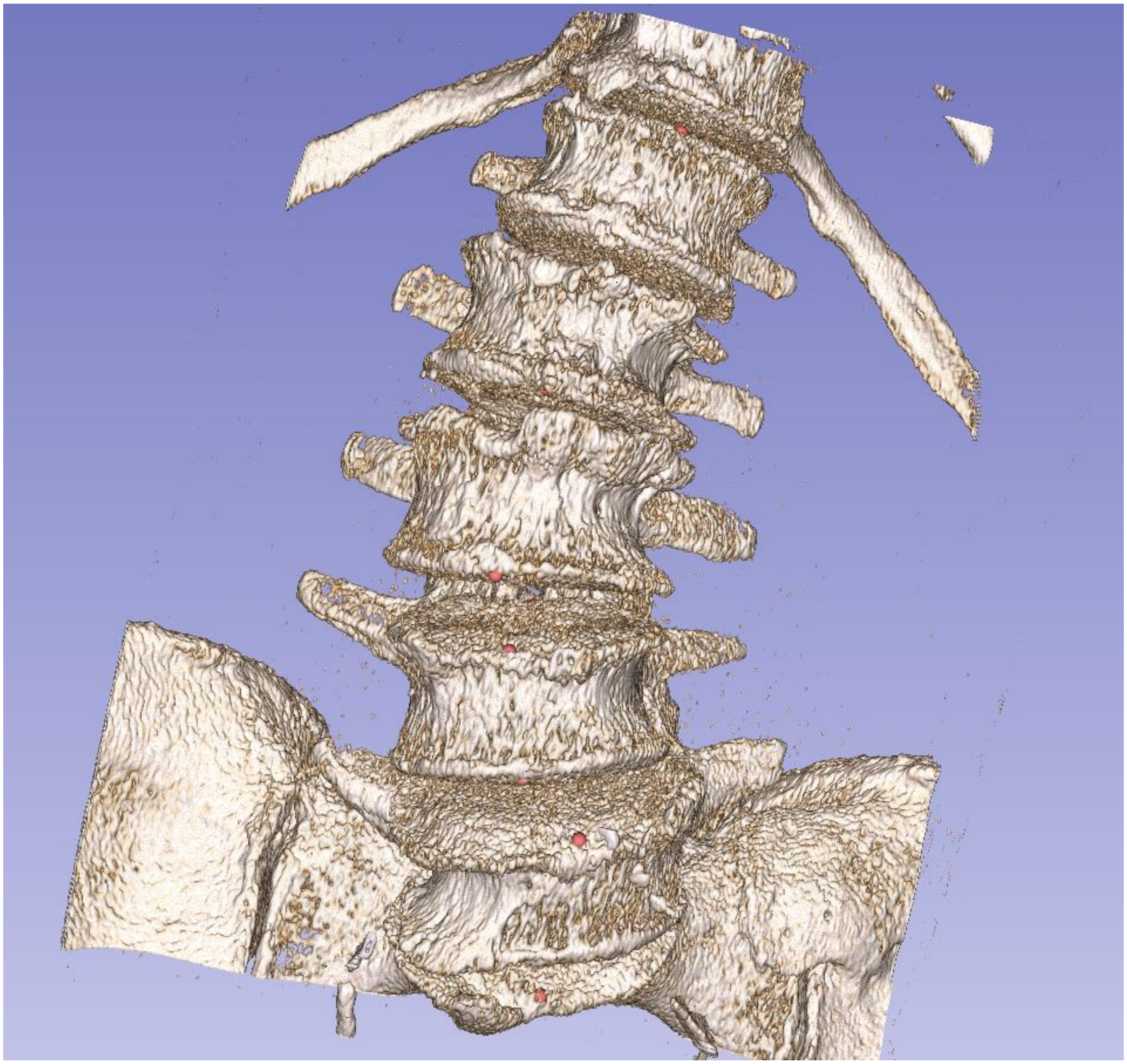

References

1. Fedorov A, Beichel R, Kalpathy-Cramer J, et al. 3D Slicer as an image computing platform for the Quantitative Imaging Network. *Magnetic resonance imaging*. 2012;30(9):1323-1341. doi:https://doi.org/10.1016/j.mri.2012.05.001

2. Shackleford JA, Shusharina N, Verberg J, et al. Plastimatch 1.6-current capabilities and future directions. 2012:

3. Andersson G, Schultz A, Nathan A, Irstam L. Roentgenographic measurement of lumbar intervertebral disc height. *Spine*. 1981;6(2):154-158.

4. Saraste H, Brostrom LA, Aparisi T, Axdorph G. Radiographic measurement of the lumbar spine. A clinical and experimental study in man. *Spine*. 1985;10(3):236-241. NOT IN FILE.

5. Frobin W, Brinckmann P, Biggemann M, Tillotson M, Burton K. Precision measurement of disc height, vertebral height and sagittal plane displacement from lateral radiographic views of the lumbar spine. *Clinical Biomechanics*. 1997;12:S1-S63.
