# Supplementary material for: Reference Data for Diagnosis of Spondylolisthesis and Disc Space Narrowing Based on NHANES-II X-rays
Source: Bioengineering (Basel). 2024 Apr 8;11(4):360. doi: 10.3390/bioengineering11040360 (PMC11048070; doi:10.3390/bioengineering11040360)
Supplement: Supplementary file 1 [file bioengineering-11-00360-s001.zip › File S5 - descriptive stats.pdf]

Supplementary File S5: Descriptive statistics and prevalence of abnormalities in the NHANES-II lumbar x-rays.

TABLE 1: Descriptive statistics for Anterior disc heights (ADH), before and after [in brackets] trimming. Trimming excluded all discs with definitive osteophytes/sclerosis or where the average disc height, disc angle, anterior sagittal plane offset (ASPO), posterior sagittal plane offset (PSPO), disc area, endplate width ratio, or superior height/width ratio was outside of the 95% confidence intervals established when all data were included.

Level	N	Mean	StdDev	CV	Median	Skewness	Kurtosis
	7175	25.43	4.5	0.18	25.57	-0.38	4.15
**L1L2**	[3835]	[26.09]	[3.18]	[0.12]	[26.07]	[0.08]	[2.58]
	7369	28.79	4.96	0.17	29.16	-0.69	4.43
**L2L3**	[3565]	[29.97]	[3.33]	[0.11]	[29.97]	[0]	[2.58]
	7374	32.07	5.32	0.17	32.46	-0.72	4.45
**L3L4**	[3436]	[33.39]	[3.6]	[0.11]	[33.38]	[-0.03]	[2.67]
	7290	36.76	7.07	0.19	37.67	-0.8	4.05
**L4L5**	[3776]	[39.19]	[4.31]	[0.11]	[39.27]	[-0.07]	[2.68]
	6836	41.52	9.4	0.23	42.39	-0.42	3.04
**L5S1**	[4315]	[44.26]	[6.39]	[0.14]	[44.41]	[-0.08]	[2.45]

Table 2: Descriptive statistics for Posterior Disc Heights (PDH), before and after [in brackets] trimming.

Level	N	Mean	StdDev	CV	Median	Skewness	Kurtosis
	7175	17.61	3.12	0.18	17.56	0.1	3.68
**L1L2**	[3835]	[18.08]	[2.57]	[0.14]	[18]	[0.13]	[2.79]
	7369	17.07	3.69	0.22	17.1	-0.11	3.45
**L2L3**	[3565]	[18.11]	[2.84]	[0.16]	[18.03]	[0.09]	[2.74]
	7374	17.47	3.95	0.23	17.58	-0.16	3.31
**L3L4**	[3436]	[18.72]	[3.04]	[0.16]	[18.67]	[0.03]	[2.81]
	7290	17.44	4.61	0.26	17.73	-0.22	3.11
**L4L5**	[3776]	[18.69]	[3.52]	[0.19]	[18.71]	[-0.03]	[3.02]
	6836	13.49	4.61	0.34	13.41	0.18	3.06
**L5S1**	[4315]	[14.21]	[3.97]	[0.28]	[14.11]	[0.09]	[3]

Table 3: Descriptive statistics for Average Disc Height (AvgDH), before and after [in brackets] trimming.

Level	N	Mean	StdDev	CV	Median	Skewness	Kurtosis
**L1L2**	7175 [3835]	21.52 [22.08]	3.15 [2.34]	0.15 [0.11]	21.58 [22.01]	-0.29 [0.08]	3.9 [2.39]
**L2L3**	7369 [3565]	22.93 [24.04]	3.69 [2.46]	0.16 [0.1]	23.23 [24.02]	-0.67 [0.03]	4.31 [2.41]
**L3L4**	7374 [3436]	24.77 [26.05]	4 [2.72]	0.16 [0.1]	25.11 [26.03]	-0.73 [-0.06]	4.34 [2.44]
**L4L5**	7290 [3776]	27.1 [28.94]	5.08 [3.13]	0.19 [0.11]	27.84 [28.98]	-0.84 [-0.13]	3.9 [2.53]
**L5S1**	6836 [4315]	27.5 [29.23]	5.81 [3.94]	0.21 [0.13]	28.21 [29.39]	-0.4 [-0.12]	2.94 [2.47]

Table 4: Descriptive statistics for Disc Angle (DA), before and after [in brackets] trimming.

Level	N	Mean	StdDev	CV	Median	Skewness	Kurtosis
**L1L2**	7175 [3835]	4.62 [4.73]	2.65 [2]	0.57 [0.42]	4.66 [4.72]	-0.23 [0.02]	3.8 [2.41]
**L2L3**	7369 [3565]	6.91 [6.97]	2.78 [2.22]	0.4 [0.32]	6.99 [6.97]	-0.25 [0.01]	3.73 [2.36]
**L3L4**	7374 [3436]	8.49 [8.52]	2.85 [2.26]	0.34 [0.27]	8.55 [8.49]	-0.25 [0.01]	3.84 [2.43]
**L4L5**	7290 [3776]	11.09 [11.76]	3.61 [2.76]	0.33 [0.24]	11.33 [11.83]	-0.36 [-0.1]	3.73 [2.54]
**L5S1**	6836 [4315]	16.19 [17.37]	5.34 [4.19]	0.33 [0.24]	16.49 [17.47]	-0.26 [-0.07]	3.1 [2.45]

Table 5: Descriptive statistics for Anterior Sagittal Plane Offset (ASPO), before and after [in brackets] trimming. Note that a positive ASPO informs the anterior inferior corner of the superior vertebra is anterior to the anterior superior corner of the inferior vertebra using the superior endplate of the inferior vertebra to define the coordinate system.

Level	N	Mean	StdDev	CV	Median	Skewness	Kurtosis
**L1L2**	7175 [3835]	-5.95 [-5.49]	2.39 [1.62]	-0.4 [-0.3]	-5.76 [-5.48]	-0.52 [-0.09]	4.65 [2.56]
**L2L3**	7369 [3565]	-7.33 [-6.95]	2.79 [1.91]	-0.38 [-0.27]	-7.27 [-6.93]	-0.02 [-0.04]	5.23 [2.51]
**L3L4**	7374 [3436]	-6.34 [-6.43]	3.4 [2.14]	-0.54 [-0.33]	-6.5 [-6.45]	0.78 [0.01]	6.38 [2.74]
**L4L5**	7290 [3776]	-5.49 [-6.77]	5.23 [3.12]	-0.95 [-0.46]	-6.24 [-6.88]	1.62 [0.26]	8.55 [2.98]
**L5S1**	6836 [4315]	-9.65 [-11.15]	6.34 [4.16]	-0.66 [-0.37]	-10.32 [-11.23]	1.56 [0.15]	9.41 [2.79]

Table 6: Descriptive statistics for Posterior Sagittal Plane Offset (PSPO), before and after [in brackets] trimming. Note that a positive PSPO informs the posterior inferior corner of the superior vertebra is posterior to the posterior superior corner of the inferior vertebra using the superior endplate of the inferior vertebra to define the coordinate system.

Level	N	Mean	StdDev	CV	Median	Skewness	Kurtosis
**L1L2**	7175 [3835]	2.7 [2.35]	2.12 [1.46]	0.78 [0.62]	2.57 [2.32]	0.42 [0.09]	4.48 [2.53]
**L2L3**	7369 [3565]	4.04 [3.92]	2.38 [1.6]	0.59 [0.41]	4.02 [3.9]	0.05 [0]	9.25 [2.51]
**L3L4**	7374 [3436]	4.12 [4.41]	2.96 [1.87]	0.72 [0.42]	4.35 [4.46]	-1.23 [-0.14]	8.48 [2.71]
**L4L5**	7290 [3776]	4.04 [5.22]	4.7 [2.75]	1.16 [0.53]	4.82 [5.44]	-2 [-0.39]	11.21 [3.18]
**L5S1**	6836 [4315]	6.07 [7.17]	5.71 [3.48]	0.94 [0.49]	6.85 [7.27]	-2.3 [-0.25]	13.57 [3.02]

Table 7: Descriptive statistics for Ventral disc heights (VDH), before and after [in brackets] trimming.

Level	N	Mean	StdDev	CV	Median	Skewness	Kurtosis
**L1L2**	7175 [3835]	26.46 [27.11]	4.71 [3.33]	0.18 [0.12]	26.6 [27.12]	-0.39 [0.08]	4.21 [2.61]
**L2L3**	7369 [3565]	29.93 [31.05]	5.18 [3.53]	0.17 [0.11]	30.25 [31.01]	-0.62 [0.04]	4.39 [2.6]
**L3L4**	7374 [3436]	32.63 [33.9]	5.46 [3.72]	0.17 [0.11]	33.04 [33.82]	-0.66 [-0.01]	4.38 [2.66]
**L4L5**	7290 [3776]	36.86 [39.36]	7.23 [4.45]	0.2 [0.11]	37.81 [39.43]	-0.72 [-0.03]	3.96 [2.71]
**L5S1**	6836 [4315]	41.73 [44.61]	9.64 [6.62]	0.23 [0.15]	42.61 [44.77]	-0.38 [-0.06]	3.06 [2.5]

Table 8: Descriptive statistics for Dorsal disc heights (DDH), before and after [in brackets] trimming.

Level	N	Mean	StdDev	CV	Median	Skewness	Kurtosis
**L1L2**	7175 [3835]	18.28 [18.74]	3.19 [2.64]	0.17 [0.14]	18.24 [18.65]	0.09 [0.14]	3.55 [2.79]
**L2L3**	7369 [3565]	17.73 [18.75]	3.71 [2.86]	0.21 [0.15]	17.79 [18.69]	-0.12 [0.09]	3.41 [2.73]
**L3L4**	7374 [3436]	17.82 [19.06]	3.92 [3]	0.22 [0.16]	17.95 [19.02]	-0.18 [0.03]	3.35 [2.81]
**L4L5**	7290 [3776]	17.61 [18.94]	4.61 [3.47]	0.26 [0.18]	17.93 [18.98]	-0.25 [-0.02]	3.15 [3.02]
**L5S1**	6836 [4315]	13.95 [14.82]	4.46 [3.74]	0.32 [0.25]	13.92 [14.74]	0.14 [0.1]	3.28 [2.98]

Table 9: Descriptive statistics for Mid-Plane Disc Angles (MPA), before and after [in brackets] trimming.

Level	N	Mean	StdDev	CV	Median	Skewness	Kurtosis
**L1L2**	7175 [3835]	2.6 [2.81]	3.26 [2.66]	1.25 [0.95]	2.66 [2.78]	-0.23 [0.07]	3.82 [2.93]
**L2L3**	7369 [3565]	6.27 [6.36]	3.39 [2.93]	0.54 [0.46]	6.33 [6.3]	-0.16 [0.09]	3.44 [2.72]
**L3L4**	7374 [3436]	9.14 [9.24]	3.56 [3.1]	0.39 [0.34]	9.14 [9.18]	-0.07 [0.1]	3.53 [2.93]
**L4L5**	7290 [3776]	15.04 [15.8]	4.44 [3.71]	0.3 [0.23]	15.16 [15.83]	-0.17 [0.02]	3.64 [2.92]
**L5S1**	6836 [4315]	24 [25.27]	5.71 [4.56]	0.24 [0.18]	24.3 [25.43]	-0.33 [-0.17]	3.34 [2.77]

Table 10: Descriptive statistics for Centroid Sagittal Plane Offset (CSPO), before and after [in brackets] trimming.

Level	N	Mean	StdDev	CV	Median	Skewness	Kurtosis
**L1L2**	7175 [3835]	-5.16 [-4.68]	2.52 [1.92]	-0.49 [-0.41]	-4.97 [-4.64]	-0.59 [-0.17]	4.55 [2.96]
**L2L3**	7369 [3565]	-7.4 [-7.01]	2.65 [1.96]	-0.36 [-0.28]	-7.3 [-6.97]	-0.17 [-0.1]	5.45 [3.06]
**L3L4**	7374 [3436]	-7.98 [-8.14]	3 [2.03]	-0.38 [-0.25]	-8.1 [-8.16]	0.86 [0.05]	7.08 [2.91]
**L4L5**	7290 [3776]	-8.25 [-9.29]	4.64 [2.84]	-0.56 [-0.31]	-8.98 [-9.48]	1.94 [0.39]	10.65 [3.38]
**L5S1**	6836 [4315]	4.28 [3.52]	6.13 [4.69]	1.43 [1.33]	3.92 [3.69]	1.15 [-0.29]	7.99 [3.5]

Tables 11 to 17: Percent of levels with abnormalities. This includes all levels in all lumbar spines from the NHANES-II study. Abnormalities were identified by standardized (Z) scores in units of std dev from average normal.

Table 11: Percent with disc heights > normal

Level	zADH>2	zADH>3	zPDH>2	zPDH>3	zVDH>2	zVDH>3	zDDH>2	zDDH>3
**L1L2**	5.1	1.0	3.8	0.6	5.1	0.9	3.7	0.6
**L2L3**	3.9	0.4	3.1	0.3	3.9	0.6	3.2	0.3
**L3L4**	3.4	0.4	2.8	0.4	3.9	0.5	2.7	0.5
**L4L5**	3.1	0.4	3.0	0.4	3.1	0.5	2.8	0.4
**L5S1**	3.3	0.2	3.3	0.5	3.1	0.3	3.0	0.5

Table 12: Percent with disc Heights < normal

Level	zADH<-2	zADH<-3	zPDH<-2	zPDH<-3	zVDH<-2	zVDH<-3	zDDH<-2	zDDH<-3
**L1L2**	9.2	2.8	6.1	0.9	8.9	2.8	6.0	0.9
**L2L3**	12.0	4.5	10.0	2.6	11.0	4.2	9.9	2.4
**L3L4**	12.2	4.9	11.0	2.9	11.5	4.7	11.1	3.1
**L4L5**	16.4	8.3	11.4	3.1	16.4	8.0	12.0	3.5
**L5S1**	15.3	5.7	5.4	0.4	15.2	5.5	6.7	0.6

Table 13: Percent with disc angles > normal. A large positive disc angle would be where the anterior disc height is much greater than it should be relative to the posterior disc height.

Level	zDA>2	zDA>3	zMPA>2	zMPA>3
**L1L2**	5.7	0.9	4.0	0.5
**L2L3**	4.4	0.6	3.7	0.4
**L3L4**	5.2	0.8	3.9	0.5
**L4L5**	3.0	0.5	2.7	0.4
**L5S1**	3.0	0.2	2.5	0.1

Table 14: Percent with disc angles < normal

Level	zDA<-2	zDA<-3	zMPA<-2	zMPA<-3
**L1L2**	6.7	1.7	5.8	1.4
**L2L3**	6.0	1.1	4.3	0.9
**L3L4**	5.7	1.4	4.2	0.8
**L4L5**	9.2	2.9	6.7	1.4
**L5S1**	9.5	2.3	9.3	2.1

Table 15: Percent with SPO > normal

Level	zASPO>2	zASPO>3	zPSPO>2	zPSPO>3	zCSPO>2	zCSPO>3
**L1L2**	4.2	0.8	10.8	3.7	2.4	0.4
**L2L3**	5.2	1.4	8.4	2.2	4.0	0.9
**L3L4**	8.5	3.6	5.3	1.3	7.2	3.2
**L4L5**	12.6	6.6	2.3	0.3	11.0	6.0
**L5S1**	10.4	4.5	2.5	0.2	6.2	2.7

Table 16: Percent with SPO < normal

Level	zASPO<-2	zASPO<-3	zPSPO<-2	zPSPO<-3	zCSPO<-2	zCSPO<-3
**L1L2**	11.6	4.3	4.2	0.9	9.1	3.0
**L2L3**	10.1	3.4	6.3	2.0	8.3	2.6
**L3L4**	7.6	2.1	8.7	4.0	5.5	1.3
**L4L5**	2.9	0.4	12.1	6.6	2.2	0.3
**L5S1**	2.6	0.2	9.4	4.9	3.4	0.6

Table 17: Percent with average disc height > or < normal

Level	zavgDH>2	zavgDH>3	zavgDH<-2	zavgDH<-3
**L1L2**	4.4	0.7	8.8	2.6
**L2L3**	3.6	0.4	13.1	5.5
**L3L4**	2.7	0.2	13.3	5.6
**L4L5**	2.3	0.1	16.8	8.8
**L5S1**	3.1	0.3	15.9	6.2

References

1. Hurxthal LM. Measurement of anterior vertebral compressions and biconcave vertebrae. Am J Roentgenol Radium Ther Nucl Med. Jul 1968;103(3):635-44.
2. Diacinti D, Guglielmi G. Vertebral morphometry. Radiologic clinics of North America. May 2010;48(3):561-75.
3. Frobin W, Brinckmann P, Biggemann M, Tillotson M, Burton K. Precision measurement of disc height, vertebral height and sagittal plane displacement from lateral radiographic views of the lumbar spine. Clin Biomech (Bristol, Avon). 1997;12 Suppl 1:S1-S63.
4. Keynan O, Fisher CG, Vaccaro A, Fehlings MG, Oner FC, Dietz J, et al. Radiographic measurement parameters in thoracolumbar fractures: a systematic review and consensus statement of the spine trauma study group. Spine (Phila Pa 1976). 10.1097/01.brs.0000201261.94907.0d doi ;00007632-200603010-00025 pii Mar 1 2006;31(5):E156-65.
5. Quint DJ, Tuite GF, Stern JD, Doran SE, Papadopoulos SM, McGillicuddy JE, et al. Computer-assisted measurement of lumbar spine radiographs. Academic radiology. 1997;4(11):742-52.
6. Brinckmann P, Frobin W, Biggemann M, Hilweg D, Seidel S, Burton K, et al. Quantification of overload injuries to thoracolumbar vertebrae and discs in persons exposed to heavy physical exertions or vibration at the work-place: The shape of vertebrae and intervertebral discs—study of a young, healthy population and a middle-aged control group. Clinical Biomechanics. 1994;9:S3-S83.
